# Investigation into the effect of skin tone modulators and exogenous stress on skin pigmentation utilizing a novel bioengineered skin equivalent

**DOI:** 10.1002/btm2.10415

**Published:** 2022-09-26

**Authors:** Kirsty Goncalves, Paola De Los Santos Gomez, Lydia Costello, Lucy Smith, Hugh Mead, Amy Simpson, Stefan Przyborski

**Affiliations:** ^1^ Department of Biosciences Durham University Durham UK; ^2^ Reprocell Europe Ltd Glasgow UK

**Keywords:** 3D cell culture, epidermal structure, in vitro, melanocytes, pigmentation, skin model, supranuclear cap

## Abstract

Human skin equivalents (HSEs) are a popular technology due to limitations in animal testing, particularly as they recapitulate aspects of structure and function of human skin. Many HSEs contain two basic cell types to model dermal and epidermal compartments, however this limits their application, particularly when investigating the effect of exogenous stressors on skin health. We describe the development of a novel platform technology that accurately replicates skin pigmentation in vitro. Through incorporation of melanocytes, specialized pigment producing cells, into the basal layer of the epidermis we are able to re‐create skin pigmentation in vitro. We observe apical distribution of melanin within keratinocytes and formation of supranuclear caps (SPNCs), only when the epidermal compartment is co‐cultured with a dermal compartment, leading to the conclusion that fibroblast support is essential for correct pigment organization. We also evaluate the commonly observed phenomenon that pigmentation darkens with time in vitro, which we further explore through mechanical exfoliation to remove a build‐up of melanin deposits in the *stratum corneum*. Finally, we demonstrate the application of a pigmented HSE to investigate drug modulation of skin tone and protection from UV‐induced damage, highlighting the importance of such a model in the wider context of skin biology.

## 1 INTRODUCTION

Human 3D skin equivalents (HSEs) are in vitro bioengineered tissues that recapitulate aspects of skin structure and function. They are extensively used to study areas of skin health including: drug discovery,^1^, ^2^ disease modeling,^3^, ^4^ exogenous stressors^5^, ^6^, ^7^, ^8^ and aging.^9^, ^10^ HSEs are advantageous over traditional 2D culture, as recreating the architecture of the tissue is essential to mimicking its function. Although HSEs provide a more physiologically relevant platform, most only contain two distinct cell types, fibroblasts and keratinocytes,^11^, ^12^ limiting their application, as appendages and supporting cell types are imperative to the complex, dynamic response of skin to stimuli.

Melanocytes are specialized, pigment producing cells located in the basal layer of the epidermis.^13^, ^14^ Melanin pigments are deposited in specialized melanosomes and transferred to adjacent keratinocytes,^15^, ^16^ accumulating in the perinuclear area as deposits termed “supranuclear caps” (SPNCs), which protect DNA from UV‐induced damage.^17^, ^18^ Understanding the role of melanins in health and disease is important not only due to their roles in photoprotection, solar lentigines^19^, ^20^ and skin cancers,^21^, ^22^ but also due to their role in many pigmentary disorders.^23^, ^24^

Although HSEs are now widely used, few models include supporting cell types such as melanocytes. This is disadvantageous as basic HSEs lack innate cellular protection provided by melanins, therefore, their response to external stimuli such as ultraviolet radiation (UVR) is not representative of in vivo tissue. There is need for a more complex, physiologically relevant HSE, which can be used as a robust and reproducible platform technology for a greater range of applications, particularly when investigating exposome impact, as exogenous stressors, including UVR and airborne pollution, are known to induce pigmentary changes.^25^, ^26^

Although a limited range of melanocyte‐containing HSEs are available, existing systems are limited, by modeling the epidermal compartment only,^27^, ^28^, ^29^, ^30^, ^31^ use of exogenous and often animal derived extracellular matrix (ECM)^32^, ^33^ or lacking ultrastructural characterization completely.^34^ These factors limit their predictive value and physiological relevance through only partially modeling the skin's microenvironment, microanatomy and ultimately function.

While epidermal models offer a reductionist approach, the absence of a dermal compartment can impact experimental outcomes, as complex cellular interactions between the two compartments are lost. This is particularly important for melanocytes, as fibroblast interaction has been reported to impact melanocyte physiology and skin pigmentation.^35^, ^36^ Dermo‐epidermal cross‐talk is thought to affect pigmentation through secretion of soluble factors by fibroblasts^37^ such as neurogulin‐1,^38^ *Dikkopf*‐1^39^ and keratinocyte growth factor (KGF).^40^ Furthermore, contributions of dermal fibroblasts to basement membrane formation^41^ and local ECM proteins^42^ have also been reported to affect melanocyte function. For this reason, it is essential to consider both the epidermal and dermal compartments simultaneously.

Previously, we have reported the development of a novel, robust, full‐thickness (FT) HSE and its thorough characterization in comparison to human skin.^43^ Here, we outline modifications to this method, incorporating human melanocytes into the basal layer of the epidermis to produce a uniformly pigmented HSE. We have conducted a thorough ultrastructural characterization of melanosome distribution and consider the key role of fibroblasts in the regulation and distribution of melanin.

We also investigate the role of desquamation in melanin turnover, which, along with autophagy, has been reported to be integral to skin tone homeostasis.^44^, ^45^ Furthermore, we describe the application of this novel pigmented HSE to study the effects of stimuli including drugs and UVR on skin tone. This demonstrates model functionality and its responsiveness to known pigmentation modulators, highlighting potential future applications including the screening of cosmetics and investigations into the effects of UV‐damage and photoageing on skin health.

## 2 RESULTS

### 2.1 Development of pigmented skin equivalents

**FIGURE 1:**
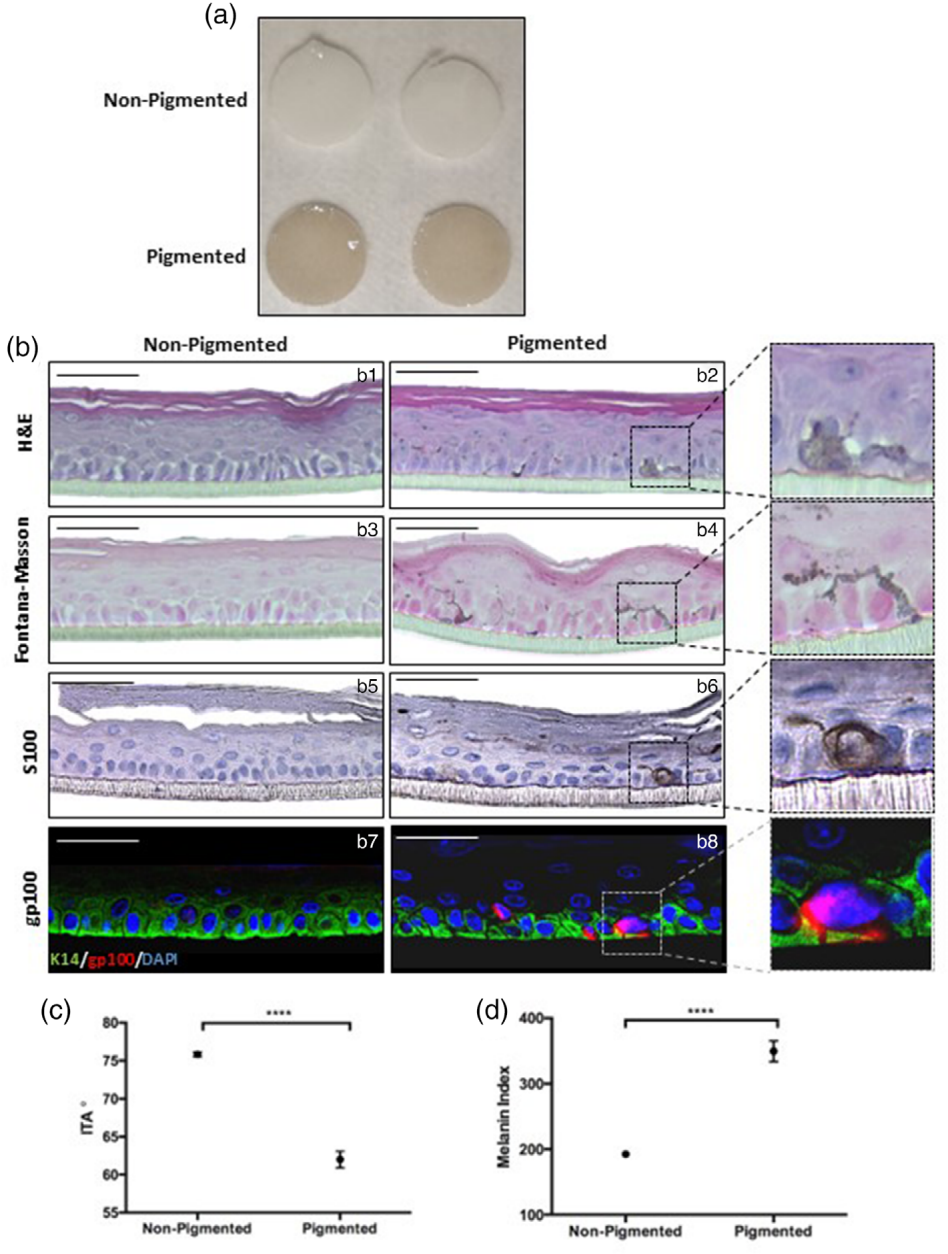
Inclusion of melanocytes produces a pigmented epidermal equivalent. Gross appearance of non‐pigmented and pigmented epidermal models (a), two examples shown, diameter 0.6 mm). Histological analysis of epidermal equivalents (b) through H&E staining (b1, b2) reveals the morphology of the epidermis and Fontana–Masson staining (b3, b4) exposes melanin distribution. Immunohistochemical analysis of S100 (b5, b6) and immunofluorescence of gp100 (b7, b8), melanocyte markers identify the location of melanocytes in the *stratum basale*. Keratin‐14 is stained in green highlights basal keratinocytes, gp100 is stained red and DAPI highlights nuclei blue. ITA (c) was significantly reduced thus darker skin tone in pigmented models and similarly a significantly greater melanin index (d) in pigmented models. Data represent mean ± SEM, *n* = 6, ****p < 0.0001. Scale bars: 50 μm.

Initially, we optimized melanocyte inclusion in a simplified epidermal‐only skin equivalent (EO‐HSE), adapted from a previously described methodology.^43^ Pigmented epidermal models appear darker (Figure 1a) and histological analysis reveals pigmented cells in the *stratum basale* (Figure 1b: b1, b2). Expression of two melanocyte biomarkers: S100 (Figure 1b: b5, b6) and gp100 (Figure 1b: b6, b7), confirm their presence. Of note, a small minority of gp100 positive melanocytes were identified within the suprabasal layers of the epidermis. Fontana–Masson staining highlights melanin deposition (Figure 1b: b3, b4) diffusely throughout the epidermis, suggesting melanin transfer to neighboring keratinocytes has occurred.

Individual typology angle (ITA) is a spectrophotometric measurement of skin tone, dependent upon melanin index (MI), that allows for a more reliable classification than conventional Fitzpatrick classification^46^ and is routinely measured in a wide range of dermatology studies.^47^, ^48^ ITA of pigmented models (Figure 1c) was significantly lower and MI (Figure 1d) significantly higher indicating a darker skin tone.

To develop a more complex and physiologically relevant HSE, we applied the optimized parameters to our previously described FT‐HSE methodology.^43^, ^49^ This HSE is engineered utilizing a porous polystyrene scaffold populated with dermal fibroblasts that secrete endogenous ECM upon which an epidermis is constructed.

**FIGURE 2:**
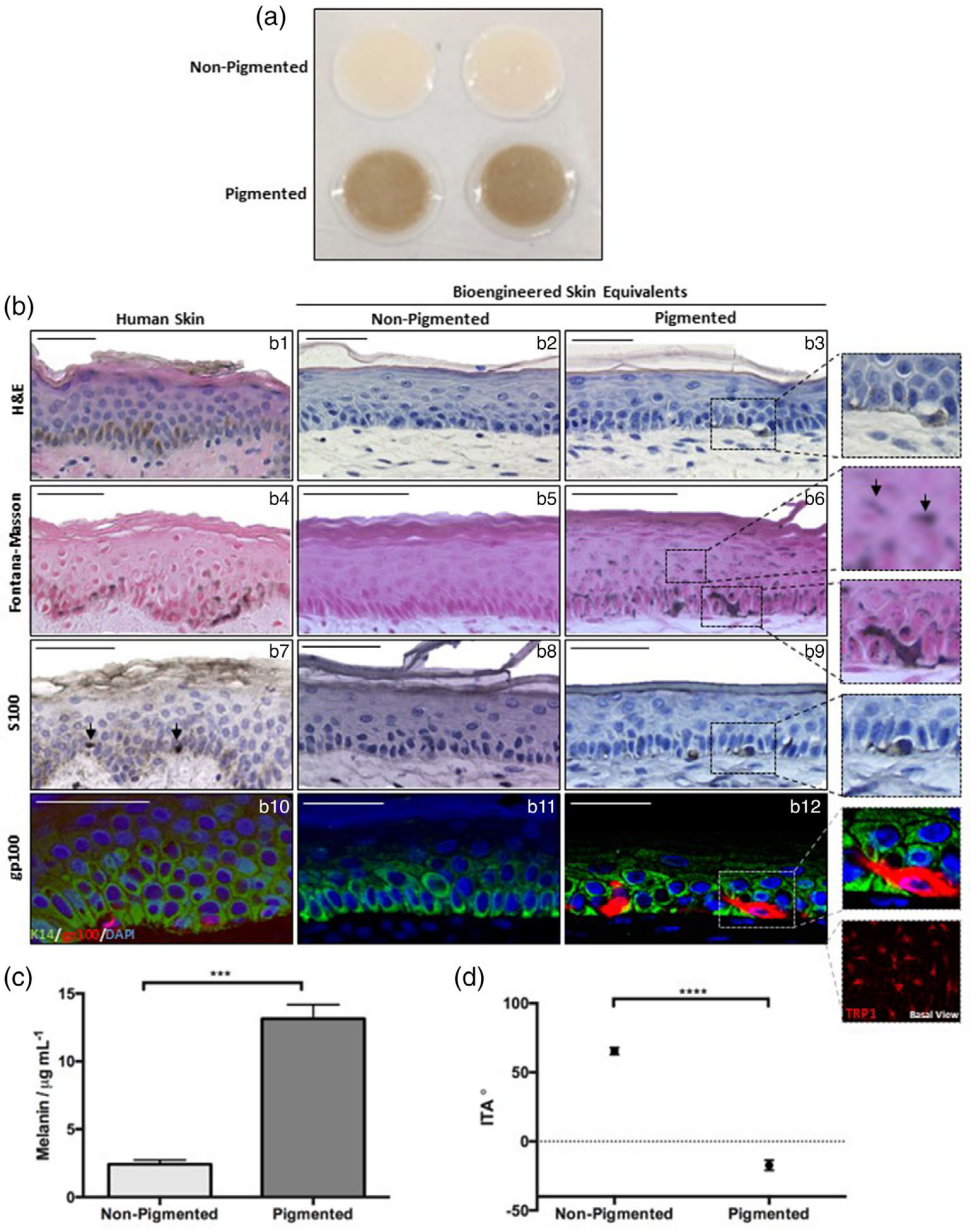
Incorporation of melanocytes into full‐thickness bioengineered skin produces a pigmented construct. Gross appearance of non‐pigmented and pigmented full‐thickness skin models (a), two examples shown, diameter 12 mm). Histological analysis of skin models in comparison to human skin (b) through H&E staining (b1–b3) and Fontana–Masson staining (b4–b6). Immunological analysis of melanocyte markers S100 (b7–b9) and gp100 (b10–b13) reveal the presence of melanocytes. Keratin‐14 is stained in green highlights basal keratinocytes, gp100 is stained red and DAPI highlights nuclei blue. A view of the *stratum basale* confirms melanocyte distribution (insert, TRP1, red). Quantification of melanin content per model (c) (data represent mean ± SEM, *n* = 6) demonstrates large quantities of melanin within the pigmented skin equivalent. Similarly ITA (d) (data represent mean ± SEM, *n* = 18) is significantly reduced in pigmented models indicating darker skin tone. ***p < 0.001, ****p < 0.0001. Scale bars: 50 μm.

Inclusion of melanocytes resulted in a uniformly pigmented HSE (Figure 2a). Histology (Figure 2b: b1–b3) revealed an organized epidermis upon a fibroblast rich dermal compartment, comparable to the structure of human skin. Melanocytes are absent within the non‐pigmented HSE but visible within the *stratum basale* of pigmented HSEs. Unlike the EO‐HSE, suprabasal melanocytes were undetected within the FT‐HSE system, supporting the correct anatomy in line with their distribution in human skin. Fontana–Masson staining (Figure 2b: b4–b6) reveals melanin distribution in the epidermis of human skin, rich in the *stratum basale* with SPNCs visible within keratinocytes. Melanin is not visible in non‐pigmented HSEs. However, within pigmented HSEs melanin deposits are present in both melanocytes and keratinocytes, clustering above the nucleus akin to SPNCs observed in native human skin.

Melanocyte presence was confirmed by expression of established biomarkers S100 (Figure 2b: b7–b9) and gp100 (Figure 2b: b10–b13) in human skin and pigmented HSEs but not within non‐pigmented HSEs. Separation of the epidermis from the dermis and subsequent immunodetection of melanocytes (TRP1) enables visualization of melanocyte distribution, revealing a uniform density of melanocytes across the whole area of the epidermis.

These data, in addition to Fontana–Masson staining of melanin distribution, indicate that melanocytes are correctly located, produce and transfer melanin to neighboring keratinocytes, forming a pigmented tissue comparable to native skin.

Melanin quantification (Figure 2c) reveals significantly increased content (5‐fold) in pigmented HSEs. ITA (Figure 2d) is significantly lower in pigmented HSEs (−30°) than non‐pigmented HSEs (60°) indicating a darker skin tone. Based on a classification described by del Bino and Bernerd,^50^ the non‐pigmented HSE is comparable to “very light” human skin whereas the pigmented HSE would be categorized as “brown” to “dark.”

### 2.2 A solid dermal foundation is required for physiological melanin distribution

Correct melanin distribution is essential for photoprotection, as SPNCs protect keratinocyte DNA from UV‐induced damage.^51^ To better examine melanin distribution within our system, we performed ultrastructural analyses.

**FIGURE 3:**
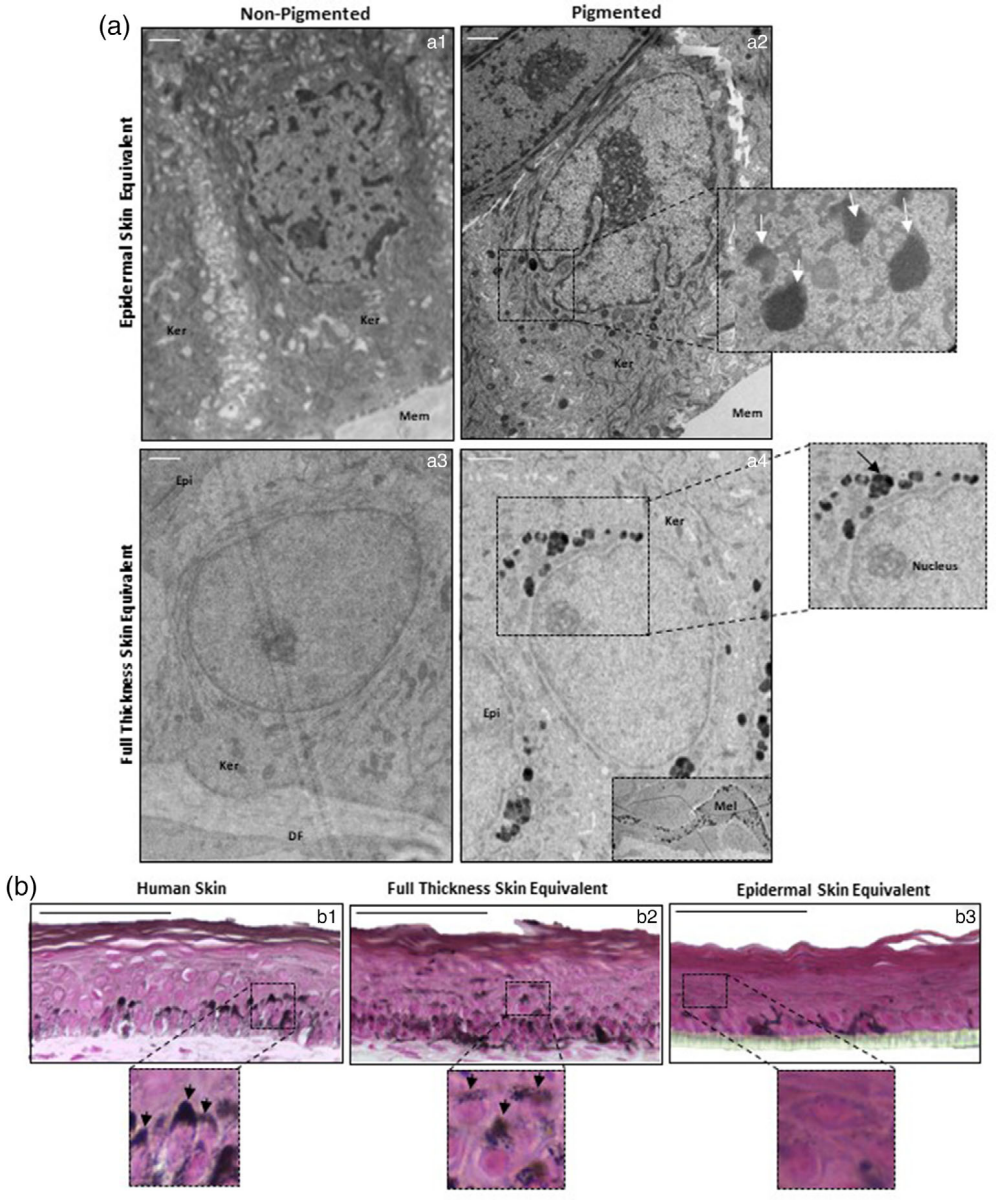
Fibroblast support is essential for correct melanin localization and supranuclear cap formation. Transmission electron micrographs (a) of non‐pigmented and pigmented epidermal‐only (a1, a2) and full‐thickness bioengineered skin equivalents (a3, a4). Melanosomes (arrows) are present within keratinocytes (Ker) in pigmented models. Melanocytes (Mel) are identifiable as cells‐rich in melanosomes within the epidermis (Epi), and were located apically to either the culture membrane (Mem) in the epidermal equivalent or a dermal compartment populated by fibroblasts (DF) in the full‐thickness skin equivalent. Fontana–Masson staining (b) highlights melanin distribution and reveals supranuclear caps in both human skin (b1) and full‐thickness skin equivalent (b2) but diffuse melanin staining without clear structures in epidermal equivalent (b3) Scale bars: (a) 5 μm (b) 50 μm.

Transmission electron microscopy (TEM) of EO‐HSE (Figure 3a: a1, a2) and FT‐HSEs (Figure 3a: b3, b4) reveals electron dense melanin deposits within pigmented models. There was no clear organization of melanin within keratinocytes of EO‐HSEs whereas in FT‐HSEs melanin is isolated to the area immediately apical to the nucleus, forming typical SPNCs as described by Tobin, 2006.^52^

Fontana–Masson staining reveals apical distribution of melanin within native human skin (Figure 3b: b1) and FT‐HSEs (Figure 3b: b2) with clear SPNCs visible within keratinocytes. This was not observed within EO‐HSEs (Figure 3b: b3), with melanin dispersed throughout the epidermis. Accordingly, we hypothesize that fibroblast support is essential for the formation of SPNCs in vitro impacting susceptibility to UV‐induced damage.

### 2.3 Desquamation is major regulatory process involved in melanin turnover in vitro

A well‐accepted limitation of the use of pigmented HSEs is that they darken with time, thought to be due to impaired desquamation in vitro.^53^ To better characterize our model, we investigated this in our system.

**FIGURE 4:**
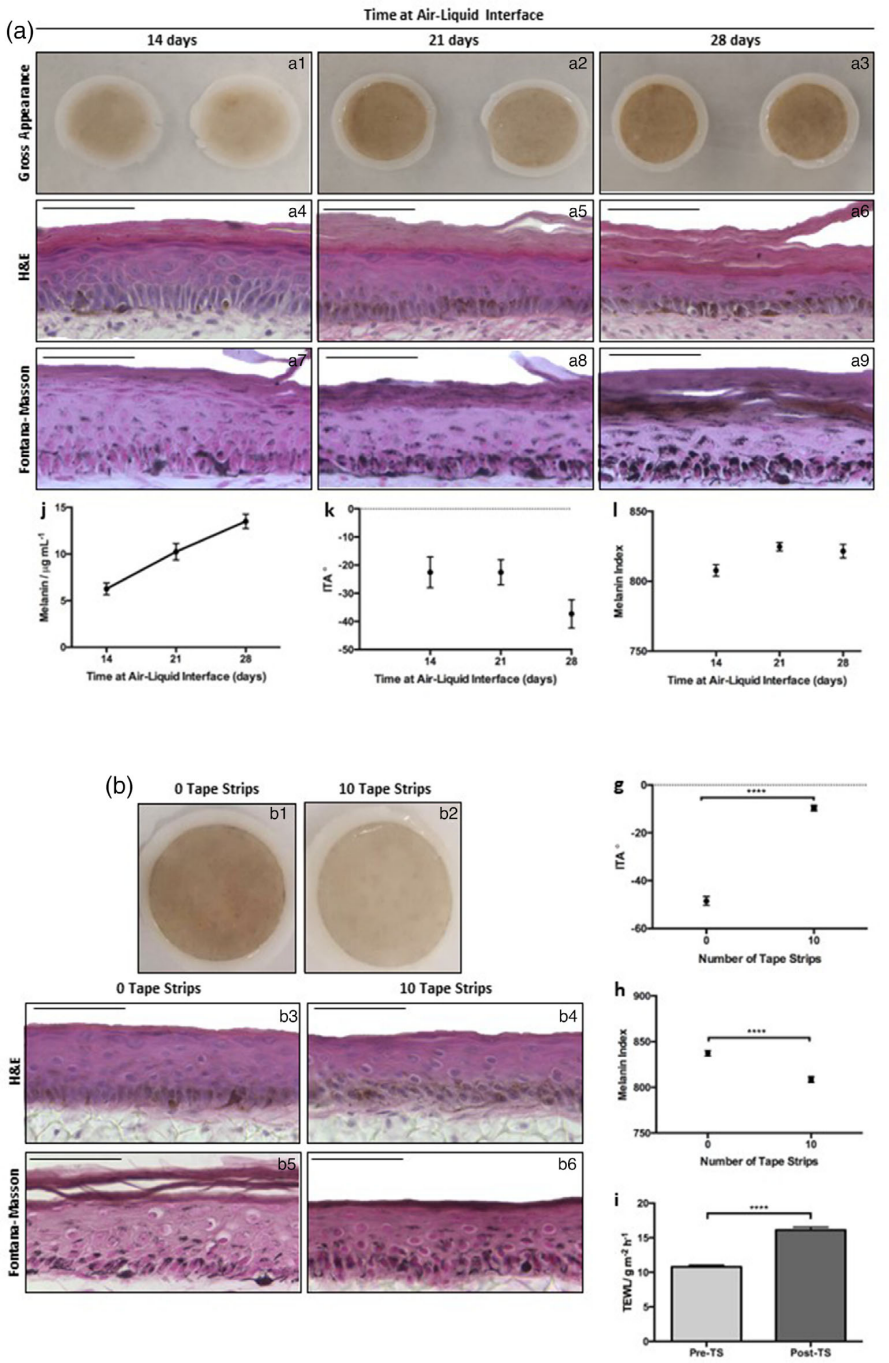
Melanin accumulates over time in culture due to a lack of desquamation in vitro. Skin equivalents darken with culture time (a: a1–a3, two examples shown, diameter 12 mm). H&E (a: a4–a6) demonstrates structure while Fontana–Masson (a: a7–a9) reveals melanin distribution. Melanin quantification (a: a10) (data represent mean ± SEM, *n* = 6) and reduced ITA (a: a11) (data represent mean ± SEM, *n* = 9) support skin darkening. Removal of *stratum corneum* (b) counteracts melanin accumulation. Gross appearance of models at 14 days ALI following 0 (b: b1) or 10 tape strips (b: b2, diameter 12 mm). H&E confirms epidermal structure (b: b3, b4) and Fontana–Masson (b: b5, b6) reveals melanin distribution. ITA (B: b7) is increased and MI (b: b8) is reduced while TEWL increased following tape stripping (b: b9) (data represent mean ± SEM, *n* = 6) ****p < 0.0001. Scale bars: 50 μm.

Over time the appearance of HSEs darkens (Figure 4a: a1–a3) and H&E staining (Figure 4a: a4–a6) demonstrates thinning of the viable epidermis at 28 days ALI with the *stratum corneum* becoming notably thicker. Melanin deposition increased throughout the suprabasal layers and concentrated in the *stratum corneum* (Figure 4a: a7–a9). This was supported by melanin quantification (Figure 4a: a10) which increased with culture time. ITA levels (Figure 4a: a11) decreased significantly at 28 days ALI (−20° to −40°), indicating skin tone darkening and MI (Figure 4a: a12) increased sharply at 21 days ALI plateauing between 21 and 28 days ALI.

To investigate the role of desquamation in this process, we utilized tape stripping, a common dermatological technique, to remove layers of the upper *stratum corneum*.^54^ Gross appearance of HSEs (Figure 4b: b1, b2) was significantly lighter following 10 tape strips. H&E staining (Figure 4b: b3, b4) revealed that the epidermal structure was unaffected by the process, further demonstrating the robustness of the skin models. Fontana–Masson suggests reduced melanin content (Figure 4b: b5, b6) following 10 tape strips. ITA (Figure 4b: b7) was significantly increased and MI decreased (Figure 4b: b8) thus supporting skin tone lightening. Transepidermal water loss (TEWL) was significantly increased following tape stripping (Figure 4b: b9), demonstrating sufficient *stratum corneum* removal to reduce barrier function.

### 2.4 Bioengineered skin equivalents respond to pigment modulating stimuli

**FIGURE 5:**
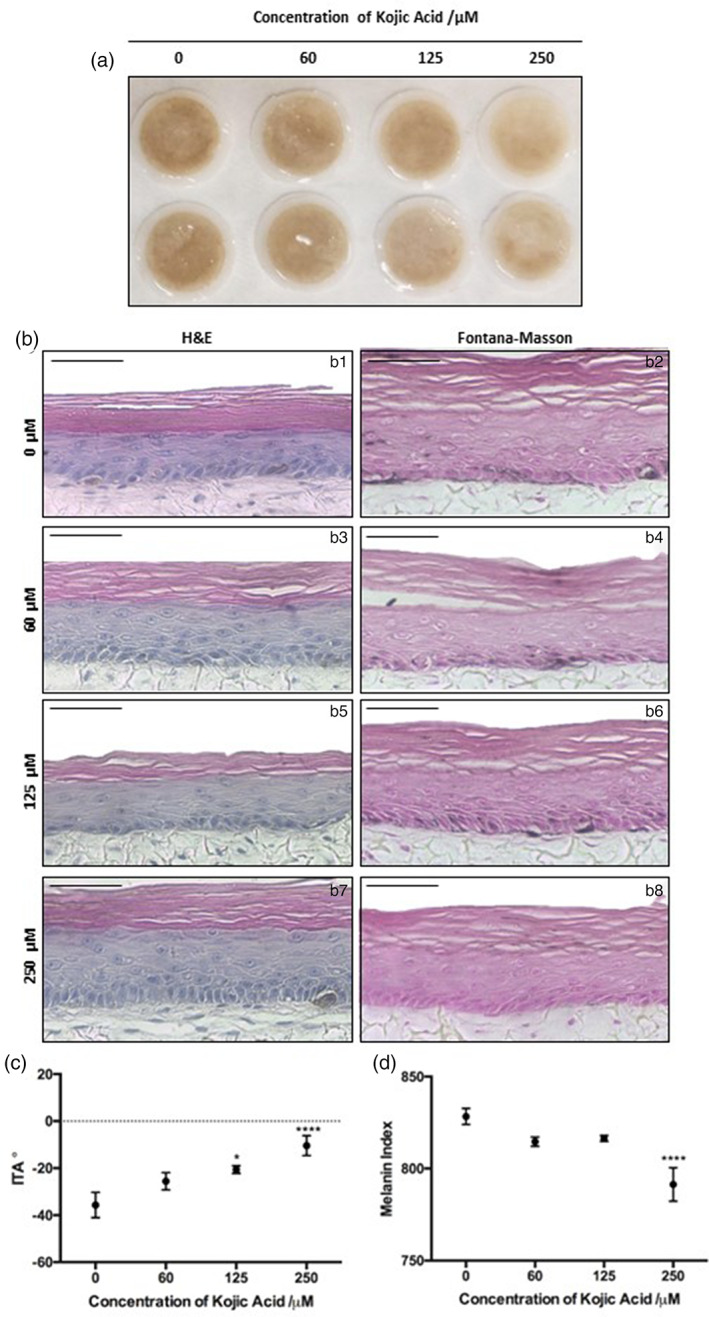
Kojic acid lightens skin tone in bioengineered skin equivalents. Gross appearance of pigmented HSEs treated with 0–250 μM Kojic acid added to their culture medium for 10 days (a, two examples shown, diameter 12 mm). Histology (b) reveals epidermal structure (b: b1, b3, b5, b7) remains unaltered despite KA treatment, while Fontana–Masson stains (b: a2, a4, a6, a8) less visible melanin staining following KA treatment. ITA (c) increases, while MI (d) decreases with high concentration treatment, demonstrating a lightening in skin tone and reduction in melanin (data represent mean ± SEM, *n* = 6). Statistical significance described compared with untreated control *p ≤ 0.05, ****p < 0.0001. Scale bars: 50 μm.

Kojic acid (KA) is a well‐characterized active commonly used in skin care regimes that lightens skin tone through inhibition of tyrosinase, the rate‐limiting enzymatic step in melanogenesis. HSEs treated for 10‐days with KA demonstrated a gross lightening effect, most notably with 250 μM KA (Figure 5a). H&E staining (Figure 5b: b1, b3, b5, b7) revealed a well‐organized epidermis following all treatment conditions, suggesting KA treatment was well tolerated and resulted in no cytotoxic effects. Fontana–Masson staining (Figure 5b: b2, b4, b6, b8) confirmed gross observations with little discernible melanin after treatment with 250 μM KA. These observations were supported by increased ITA (Figure 5c) and reduced MI (Figure 5d), indicating significant skin tone lightening.

**FIGURE 6:**
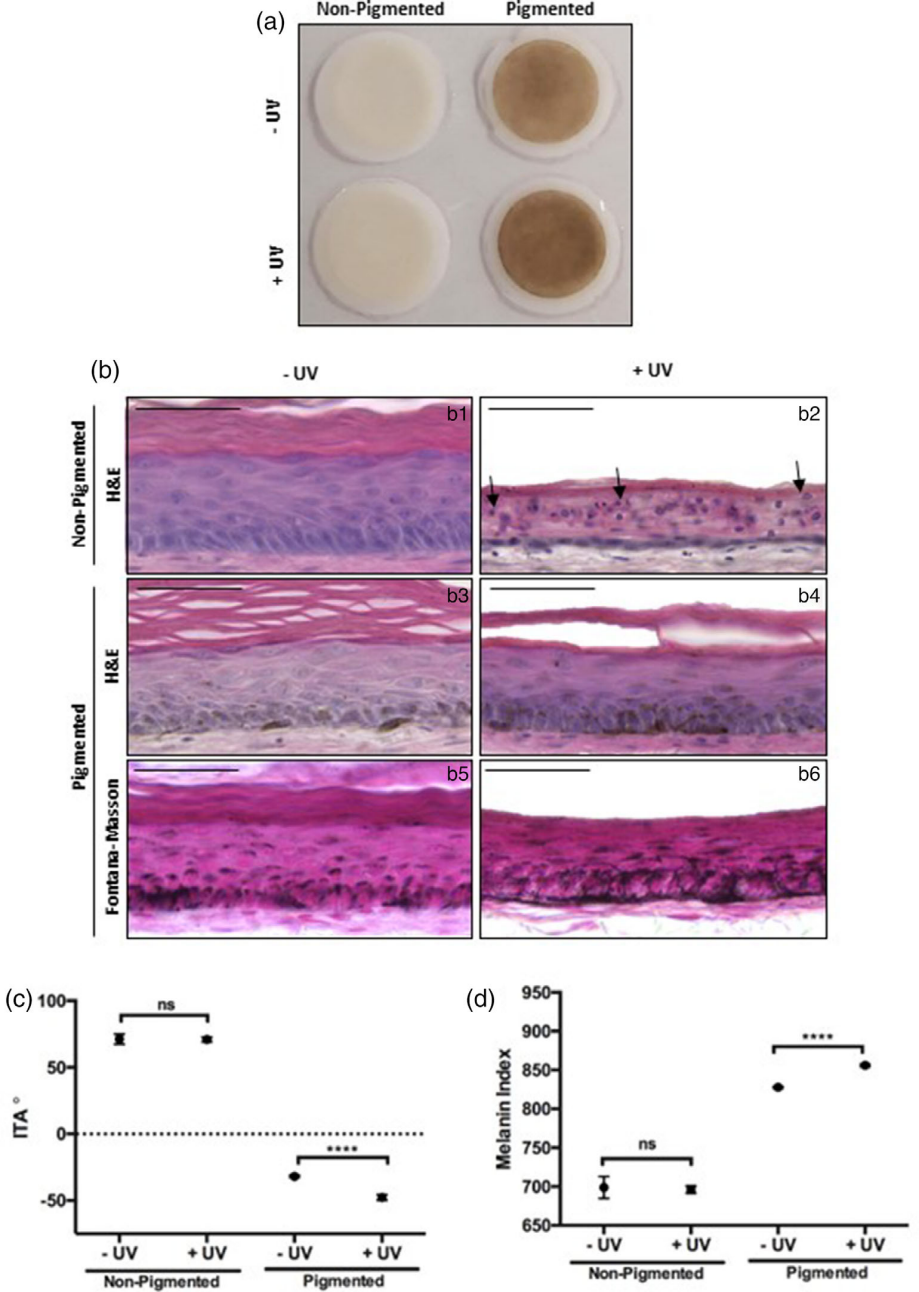
Melanin provides innate protection from UV‐induced damage in vitro. Gross appearance of non‐pigmented and pigmented skin equivalents either non‐irradiated (−UV) or irradiated 5 times with 3.3 J cm^−2^ (96.5% UVA, 3.5% UVB) (+UV) and harvested 48 h following exposure (a, diameter 12 mm). H&E (b) reveals epidermal structure (b: b1–b4) and Fontana–Masson (b: b5, b6) exposes melanin content of pigmented models. The epidermis of UV irradiated non‐pigmented models is apoptotic with formation of “sunburn cells” (arrow heads), whereas epidermal structure is unaltered in pigmented models following UV irradiation. ITA (c) and melanin index (d) remain unchanged in non‐pigmented models, whereas they decrease and increase, respectively, following UV exposure in pigmented models, indicating tanning (data represent mean ± SEM, *n* = 9). ****p < 0.0001. Scale bars: 50 μm.

UVR is a potent pigment‐inducing stimulus and has previously been demonstrated to induce hyperpigmentation in bioengineered skin equivalents.^55^ Gross appearance of HSEs (Figure 6a) either non‐irradiated or irradiated five times with 3.3 J cm^−2^ (96.5% UVA, 3.5% UVB), showed pigmented HSEs became notably darker, displaying a “tanning” effect. Histological analysis of epidermal structure (Figure 6b) revealed UV‐irradiated non‐pigmented HSEs contained apoptotic keratinocytes with condensed nuclei characteristic of “sunburn cells”.^56^, ^57^, ^58^ Additionally, the epidermis was significantly thinner than their non‐irradiated counterpart, which displayed a stratified, organized epidermis, suggesting UV‐induced apoptosis has had a significant impact on epidermal structure.^59^, ^60^ Tissue damage was not observed in UV‐irradiated pigmented HSEs; there was no observable apoptotic cells and epidermal structure remains unaffected, suggesting pigmented HSEs are well protected from UV‐induced damage, supporting previous findings in epidermal‐only systems.^8^

Increased melanin deposition in UV‐irradiated HSEs was evident (Figure 6b: b5, b6). ITA (Figure 6c) and MI (Figure 6d) remained unchanged in non‐pigmented UV‐irradiated HSEs but there was a significant reduction in ITA (skin tone darkening) and an increase in MI following UV‐exposure in pigmented HSE. While this further confirmed the protective nature of melanins, this also demonstrated that melanogenesis can be stimulated through UV exposure and HSE ability to react to stimuli in a similar manner to native human skin.

## 3 DISCUSSION

Bioengineered HSEs are becoming increasingly popular due to limitations on animal testing for cosmetic use.^61^ However, many FT‐HSEs contain only two basic cell types to recapitulate distinct epidermal and dermal compartments. Although this may be sufficient for some applications, investigations into the impact of exogenous stressors on skin health require a more complex system. Herein, we describe the development and in‐depth characterization of a pigmented FT‐HSE that recapitulates both the structure and distribution of melanin throughout the epidermis. We also demonstrate functionality through its responses to known stimuli including a potent skin lightening agent and UVR.

The formation of melanin SPNCs within keratinocytes is a well‐known phenomenon that protects nuclear DNA from UV‐induced damage.^62^ A modest number of pigmented HSEs have been reported in the literature, however they lack extensive characterization, with limited evidence provided relating to the formation of SPNCs.^32^, ^34^, ^41^, ^63^, ^64^, ^65^ To our knowledge, presence of SPNCs in vitro has only been reported by Gibbs et al.^66^ in a reconstructed epidermal system requiring patient‐derived tissue for construction. Although the use of patient‐derived tissue can lead to personalized medicine applications, the availability of tissue can be a limiting factor in the creation of such models, restricting their use for industrial pursuits and large scale applications. In this study, we describe the creation of a pigmented HSE from commercial cell sources that is both robust and reproducible for industrial and academic pursuits that can be scaled up for high throughput applications.

We hypothesized that the formation of SPNCs and correct localization of melanocytes to the *stratum basale* relies on dermo‐epidermal interactions. This is due to the observation that SPNCs only form in FT‐HSEs as opposed to EO‐HSEs. This notion is supported by well‐established links between dermo‐epidermal interactions and gross pigmentation; proposed mechanisms include secretion of fibroblast‐derived soluble factors,^67^ direct physical interaction with the basement membrane,^41^ biochemical interactions with ECM components^68^ along with direct biophysical influences of the culture substrate. Further investigations are required to elucidate the specific mechanism, or mechanisms, that underpin dermal influence on epidermal pigmentation in this system, however these data do support the requirement of a robust dermal formation to recapitulate accurate pigmentation in vitro.

In this study, we have demonstrated not only the correct localization of melanin, but also functionality. When exposed chronically to UVR, we found that pigmented HSEs remained intact with healthy epidermal structure, whereas non‐pigmented HSEs became apoptotic and displayed characteristic sunburn damage.^56^ We also observed a tanning effect caused by increased melanogenesis within UVR‐exposed pigmented HSEs, demonstrating that our engineered HSE reacts to stimuli in‐line with the known physiological response. We postulate that this innate protection offered by melanin prevents damage to the epidermis in pigmented HSEs, mediated by the formation of protective SPNCs.

Autophagy has often been considered the driving factor in epidermal melanin homeostasis.^69^ However, Joly‐Tonetti et al.^45^ suggest an alternative process involving asymmetric melanosome distribution, and found no evidence of autophagy in healthy skin. Asymmetric distribution, in combination with desquamation and epidermal turnover, is hypothesized to be key in maintaining skin tone, a theory supported by findings of varying rates of desquamation in different skin tones^70^ and skin tone lightening by agents that enhance desquamation.^44^

We observed the well‐known phenomenon of increasing pigmentation over time in HSEs,^71^ and hypothesized that this was due to attenuated desquamation in vitro,^72^ as a build‐up of *stratum corneum* concentrates melanin, preventing keratinocyte turnover that occurs in vivo. To test this, we used a common dermatological technique of removing material from the *stratum corneum* surface by tape stripping.^73^ Tape stripping removed layers of the *stratum corneum* to a point where barrier function was compromised and resulted in significant skin tone lightening, supporting the finding of Joly‐Tonetti that desquamation is an important regulatory mechanism. Particularly in darker skin tones as melanin concentrates in the *stratum corneum* and demonstrated by the “darkly pigmented” melanocytes used to recapitulate pigmentation in this study.

Irregular pigmentation is a consequence of many dermatological disorders including: photoageing,^74^, ^75^ post‐inflammatory hyperpigmentation^76^, ^77^ and pigmentary changes such as melasma or senile lentigo. For this reason, many cosmetics contain agents known to modulate skin tone such as thiamidol,^78^, ^79^ niacinamide^80^ and KA.^80^ In vitro pre‐clinical assessments have become an important tool to provide insight into the effect of formulations prior to progression to clinical trials. Herein, we describe the ability of KA, a well‐characterized inhibitor of melanogenesis, to lighten the skin tone of our HSE, establishing its response to known actives and providing an example application. Although there is published literature on the use of 3D technologies to screen hypopigmenting agents,^65^, ^81^ some model systems including a commonly cited commercially available technology do not exhibit the expected response to KA.^82^ We therefore demonstrate that our HSE not only recapitulates physiologically accurate pigmentation but, crucially, responds to stimuli in a manner akin to native skin.

Through this study we have presented a well‐characterized FT‐HSE and its thorough ultrastructural analysis. Characterization to this level is lacking in many other pigmented HSEs. We describe the correct localization of melanin, formation of protective SPNCs and the ability of such structures to protect against UVR‐induced damage. Furthermore, we also demonstrate the ability of a well‐known active to reduce melanin content and lighten skin tone. These data demonstrate the ability of the HSE to respond to stimuli both by upregulation and downregulation of melanin production accordingly and in line with the expected response, validating the mechanistic integrity of melanogenesis in vitro.

These findings suggest that our pigmented HSE recapitulates both physiological structure and function of melanin within the epidermis, providing a platform technology for a vast array of research and development opportunities.

## 4 MATERIALS & METHODS

### 4.1 Cell culture

#### 4.1.1 Skin equivalent generation

Commercially available cells used to create HSEs including human neonatal keratinocytes #1817888, #1944927, #2288858 and #2018512 (HEKn, ThermoFisher Scientific, Loughborough, UK), darkly pigmented human neonatal epidermal melanocytes #2077650 (HEMn‐DP, ThermoFisher Scientific) and neonatal dermal fibroblasts #1366356 and #1366434 (HDFn, ThermoFisher Scientific). Cells were screened for infectious agents by the manufacturer and used in HSEs within three passages.

HSEs were generated as previously described.^43^, ^49^ For pigmented HSEs, HEMn‐DP were trypsinized and seeded simultaneously with HEKn onto a 28‐day matured dermal compartment at a ratio of 1:10 (melanocytes:keratinocytes). Cultures were then maintained in submerged culture for 48 h and raised to the ALI to promote keratinocyte differentiation and stratification, for a further 14‐days prior to use in experiments.

#### 4.1.2 UV exposure

Bio‐Sun UV (Vilber Lourmat, Collégien, France) was used to expose HSEs to UVR. This system has a defined spectrum including a range of wavelengths that peak at 365 nm (UVA) or 312 nm (UVB). During irradiation the sample is exposed to a mixed spectra as a result of energy levels selected for both UVA and UVB. Delivery of both spectra begins in unison, however the time of delivery completion varies according to the dose selected for each type of radiation.

A physiologically representative UVR exposure was calculated using previously reported parameters to represent northern hemisphere countries including USA and UK. A UVA:UVB ratio of 27 was selected based on the calculated average daily UV spectrum published by Christiaens et al., 2005.^83^ A terrestrial dose of 110–160 J cm^−2^ was selected representative of latitudes 30° to 60°^83^ and an average time of 10% daylight time spent outdoors assumed, which signifies average exposure of children and indoor workers.^84^, ^85^, ^86^ An outdoor condition factor of 30% was also incorporated to account for environmental features.^85^, ^86^, ^87^ This published data was collated and a representative UVR dose of 3.3–4.8 J cm^−2^ was calculated.

In this study, HSEs were irradiated every 24 h for 5 consecutive days with 3.3 J cm^−2^ (96.5% UVA, 3.5% UVB). HSEs were harvested 48 h after the final irradiation. Non‐irradiated were by washed in DPBS and left in the cell culture hood for the duration of the exposure to control for the effects of environmental fluctuations.

#### 4.1.3 Drug treatment

Kojic acid (Sigma‐Aldrich, Dorset, UK) was reconstituted in Epilife (ThermoFisher Scientific) and added to HSEs at a range of concentrations 0–250 μM. HSEs were treated at 14 ALI and treatment replaced at each media change HSEs were harvested after 10 days.

#### 4.1.4 Tape stripping

D‐Squame tape strips (CuDerm, Texas) were applied to the surface of HSEs with even pressure by use of the D500‐D‐squame pressure instrument that ensures a standardized pressure of 225 g cm^−2^. Tape strips were removed using forceps and 10‐tape strips were removed sequentially from each HSE.

### 4.2 ITA & melanin index readings

Individual typological angle (ITA) is a colorimetric measure of skin tone phenotype, through which skin tone can be classified from “very light” to “dark” tones. This offers a more reliable greater range skin tone measurements compared with well accepted Fitzpatrick scaling.^50^ ITA and MI readings were obtained using the SkinColourCatch (Delfin Technologies, Surrey, UK).

### 4.3 Transepidermal water loss measurements

Transepidermal water loss (TEWL) was measured using the VapoMeter (Delfin Technologies). HSEs were allowed to equilibrate at room temperature for 20 mins prior to measurement.

### 4.4 Human skin samples

Skin biopsies from young adult Caucasian women were collected by Procter and Gamble USA, under an IRB‐approved clinical protocol in compliance with local laws and regulations.

### 4.5 Paraffin wax embedding

Samples were fixed in 4% paraformaldehyde and dehydrated through ethanols. Samples were incubated in Histoclear (Scientific Laboratory Supplies, Nottingham, UK) alone, with molten paraffin wax (ThermoFisher Scientific) and paraffin wax alone. Samples were embedded in plastic molds (CellPath, Newton, UK) with paraffin wax and sectioned transversely using a microtome (Leica RM2125RT). 5 μm sections were placed onto charged microscope slides (ThermoFisher Scientific).

### 4.6 Hematoxylin & Eosin (H&E) staining

Samples were deparaffinized in Histoclear (Scientific Laboratory Supplies) and rehydrated through a series of ethanols. Samples were then incubated in Mayer's hematoxylin (Sigma‐Aldrich) for 5 min followed by alkaline ethanol for 30 s. Slides were dehydrated through a series of ethanols prior to incubation with eosin (Sigma‐Aldrich) for 30 s and further dehydrated. Finally, slides were cleared in Histoclear and mounted with Omnimount (Scientific Laboratory Supplies).

### 4.7 Fontana–Masson staining

Fontana–Masson melanin staining was achieved using a commercially available kit (Abcam, Cambridge, UK, ab150669) following manufacturer's instructions.

### 4.8 Immunofluorescence

Sections were deparaffinized in Histoclear and rehydrated through a series of ethanols. Antigen retrieval was performed in citrate buffer pH 6 (Sigma‐Aldrich) at 95 °C for 20 min, followed by blocking and permeabilization for 1 h with 20% neonatal calf serum (NCS, Sigma‐Aldrich) and 0.4% Triton X‐100 (Sigma‐Aldrich) in phosphate buffered saline (PBS). Samples were then incubated overnight at 4 °C in primary antibody diluted in blocking buffer (gp100, Abcam, ab137078, 1:100) (Keratin‐14, Abcam, ab7800, 1:100). Slides were washed three times in PBS and incubated with the appropriate secondary antibody diluted in blocking buffer for 1 h at room temperature (donkey anti‐rabbit Alexa Fluor 488 or donkey anti‐mouse Alexa Fluor 594, ThermoFisher Scientific, 1:1000) and washed three times in PBS. Finally, slides were mounted using Vectashield Hardset with DAPI (Vector Laboratories, Peterborough, UK).

### 4.9 Immunohistochemistry

Sections were deparaffinized in Histoclear and rehydrated through ethanols. Peroxidase blocked (3% hydrogen peroxide in methanol) for 15 min and washed in PBS. Antigen retrieval was achieved at 95 °C for 20 min in citrate buffer, before blocking (10% NCS, 0.1% BSA, 0.4% Triton‐X‐100) for 1 h. Primary antibody (S100, Abcam, ab52642, 1:100) was then applied for 1 hand washed in PBS before a biotin conjugated secondary antibody (Mouse anti‐rabbit IgG, Santa Cruz Biotechnology, California, US, sc2491, 1:200) was added for 1 h. Slides were then washed in PBS and ABC reagent (Santa Cruz Biotechnology) for 30 min before being developed in DAB reagent (Vector Laboratories). Slides were washed in dH_2_O and counterstained in Gill's Hematoxylin (Sigma‐Aldrich) before being dehydrated, cleared in Histoclear and mounted using Omnimount.

### 4.10 Epidermal whole mount staining

In order to visualize the distribution of melanocytes across the epidermis, the epidermal layer was separated from the underlying dermis through enzymatic digestion. The HSE was placed *stratum corneum* down in a solution of 500 U ml^−1^ Dispase (Sigma‐Aldrich) and incubated at room temperature for 20 min. Following this the epidermis was peeled using forceps, from the dermis and washed twice in PBS. The epidermal sample was then blocked and permeabilized for 1 h with 20% NCS and 0.4% Triton X‐100 in PBS. Samples were then incubated at room temperature for 2 h in primary antibody diluted in blocking buffer (TRP1, Abcam, ab6190709, 1:100). Samples were washed three times in PBS and incubated with the appropriate secondary antibody diluted in blocking buffer for 1 h at room temperature (donkey anti‐mouse Alexa Fluor 594) and washed three times in PBS. Epidermises were then mounted on microscope slides using Vectashield.

### 4.11 Light microscopy

Histology, Fontana–Masson and immunohistochemistry images were captured using Leica ICC50 high‐definition camera and Brightfield microscope. Immunofluorescence images were taken using the Zeiss 880 confocal microscope with Airyscan and Zen software.

### 4.12 Electron microscopy

Samples were prepared for TEM as previously described^43^ and images captured on a Hitachi H7600 TEM.

### 4.13 Melanin quantification assay

Quantification of melanin content was achieved using a previously published methodology.^88^

### 4.14 Statistical analysis

GraphPad Prism software was used to measure the statistical significance by use of a Student's *t*‐test or one‐way ANOVA with Tukey's post‐hoc as appropriate. *p ≤ 0.05, **p ≤ 0.01, ***p ≤ 0.001, ****p ≤ 0.0001.

## 5 CONCLUSIONS

In this study, we have described the development of a complex, pigmented HSE that is able to appropriately modulate skin pigmentation in response to external stimuli such as UVR and drugs. We outline the imperative nature of recapitulating physiological microanatomy in the formation of protective supranuclear caps, leading to physiological tissue function. An ultrastructural characterization of bioengineered tissues is essential to enhance our understanding of tissue function providing a platform for use with a wide range of applications.

## AUTHOR CONTRIBUTIONS

**Kirsty Goncalves:** Conceptualization (lead); data curation (lead); formal analysis (lead); investigation (lead); methodology (lead); project administration (lead); writing – original draft (lead). **Paola De Los Santos Gomez:** Data curation (equal); formal analysis (equal); investigation (equal); methodology (equal); writing – review and editing (equal). **Lydia Costello:** Data curation (supporting); formal analysis (supporting); investigation (supporting); methodology (supporting); validation (supporting); writing – review and editing (equal). **Lucy Smith:** Data curation (equal); formal analysis (supporting); writing – review and editing (equal). **Hugh Mead:** Data curation (supporting); formal analysis (supporting); investigation (supporting). **Amy Simpson:** Data curation (supporting). **Stefan Przyborski:** Conceptualization (lead); funding acquisition (lead); project administration (lead); resources (lead); supervision (lead); writing – review and editing (lead).

## CONFLICT OF INTEREST

The authors declare no conflict of interest.

### PEER REVIEW

The peer review history for this article is available at https://publons.com/publon/10.1002/btm2.10415.

## Data Availability

No datasets were generated or analyzed during the current study.
